# Distribution of Mast Cells and Locations, Depths, and Sizes of the Putative Acupoints CV 8 and KI 16

**DOI:** 10.1155/2017/2953278

**Published:** 2017-03-27

**Authors:** Sharon Jiyoon Jung, Haim Song, Yu Yeon Kim, Jungdae Kim, Sungchul Kim, Yoon-Kyu Song, Kwang-Sup Soh

**Affiliations:** ^1^Advanced Primo Research Laboratory, Advanced Institute of Convergence Technology, Seoul National University, Suwon 16229, Republic of Korea; ^2^Department of Transdisciplinary Studies, Graduate School of Convergence Science and Technology, Seoul National University, Suwon 16229, Republic of Korea; ^3^Department of Life Science, Suwon University, Suwon 18323, Republic of Korea; ^4^Pharmacopuncture Medical Research Center, Korean Pharmaceutical Institute, Seoul 07525, Republic of Korea; ^5^Department of Acupuncture & Moxibustion, Gwangju Medical Hospital, Wonkwang University, Gwangju 61729, Republic of Korea

## Abstract

The anatomical locations and sizes of acupuncture points (APs) are identified in traditional Chinese medicine by using the cun measurement method. More precise knowledge of those locations and sizes to submillimeter precision, along with their cytological characterizations, would provide significant contributions both to scientific investigations and to precise control of the practice of acupuncture. Over recent decades, researchers have come to realize that APs in the skin of rats and humans have more mast cells (MCs) than neighboring nonacupoints. In this work, the distribution of MCs in the ventral skin of mice was studied so that it could be used to infer the locations, depths from the epidermis, and sizes of three putative APs. The umbilicus was taken as the reference point, and a transversal cross section through it was studied. The harvested skins from 8-week-old mice were stained with toluidine blue, and the MCs were recognized by their red-purple stains and their metachromatic granules. The three putative APs, CV 8 and the left and the right KI 16 APs, were identified based on their high densities of MCs. These findings also imply that acupuncture may stimulate, through MCs, an immune response to allergic inflammation.

## 1. Introduction

Acupuncture has been a major medical practice for thousands of years in China, Korea, and Japan, yet the mechanism underlying acupuncture has still not been unambiguously identified on a scientific basis and, thus, needs to be investigated further. Among the findings of numerous research efforts that have investigated the mechanism underlying acupuncture, the finding that acupuncture treatment appears to improve the immune function seems particularly notable [1]. Mast cells (MCs) are active carriers of innate immunity against such conditions as allergies and inflammatory diseases [2, 3]. Therefore, the finding that the population of MCs is denser at APs and meridians than at nearby nonacupoints is not surprising [4, 5]; stimulation of the APs by using electroacupuncture or moxibustion has been found to induce degranulation of MCs [6–8]. This cytological characteristic of APs is in accordance with the recent finding that a particular tissue, called the primo node, in the primo vascular system (PVS) has a high population density of MCs [9]. The PVS was first proposed as the anatomical structure corresponding to the APs and meridians by BH Kim in the early 1960s, and it has long been thought, but not proven, to be a possible scientific foundation for Korean medicine [10].

In traditional Chinese medicine, the anatomical locations of APs can be identified by using the cun measurement method. However, dermal electrical impedance measurements do not significantly improve the precisions of those locations, and information on the depths from the epidermis and the sizes of the APs is totally lacking. Thus, in this work, we used the distributions of the MCs at three acupoints, the CV 8 and the left and the right KI 16 APs, to estimate their locations, sizes, and depths from the epidermis. Knowledge of those parameters to submillimeter precision, along with knowledge of their cytological characteristics, would provide significant contributions to scientific investigations and to precise control of the practice of acupuncture.

Apart from acupuncture and the immune response, MCs synthesize, store, and release histamine and other mediators of inflammation [11, 12] and are crucial for the maintenance of tissue homeostasis, tissue repair, and the remodeling of the extracellular matrix [13, 14]. In addition, MCs accelerate epithelial-to-mesenchymal transitions, extracellular matrix degradation, and disease progression in some carcinomas [15]. Therefore, MCs are important cytological ingredients connecting Western and Eastern medicine [16].

In Western medicine, the locations and the distributions of MCs have been studied in connection with their physiological functions. For instance, MCs are located at the host-environment interface so as to initiate the host's defenses against intruders [17]. An early quantitative study on the distribution of MCs in normal mouse skin was performed by Larsson and Sylven to address the reactions of MCs to different chemical agents [18]. They developed a fairly reliable counting technique, but their study was limited to a small area of the dorsal skin, that is, an area on each side of the spine in the interscapular regions. They found that the numbers of MCs showed left-right symmetry in each individual, but the variations in the numbers of both dermal and hypodermal MCs between individuals were so large that the determination of an average standard number was not practicable. The MCs of the skin lie in the dermis and the hypodermis, where they are grouped around blood vessels and nerves. A quantitative study on the association between MCs and blood vessels in human skin was first reported by Eady et al. [19]. They observed statistically significant correlations between MC counts and blood vessel counts in skin from the upper arm, but no similar correlations were observed in skin from the forearm. They also confirmed an uneven distribution of dermal MCs in human skin, which was consistent with the previous finding for mouse skin.

Although researchers in Western medicine noticed a highly-clumped distribution of dermal MCs with a population density that varied greatly within a small area encompassing a few mm^2^ of skin [19], they did not recognize the relation between the regions of the skin with the clumped high density of MCs and the APs. Zhu et al. pointed out that higher densities of MCs were found at some APs compared with neighboring nonacupoints [4, 5]. MCs, blood vessels, and nerves were found to gather to form a complex at APs, and acupuncture stimuli were found to cause MCs to migrate to and be recruited in the APs and the meridians [20]. However, they compared the densities of MCs in the APs with those of unspecified nonacupoints rather than showing the distribution of MCs throughout the skin. Therefore, the question of whether the densities of MCs in the skin could be used to get information on the locations, depths, and sizes of APs was not answered. Independently, the evidence for higher densities of MCs at APs was reinforced by a series of works on the abundance of MCs in the PVS in the abdominal cavity [21–23] and the abdominal wall [9]. This PVS prompted us to develop the current scheme of estimating the anatomical parameters for the APs in skin by making use of the distribution of MCs. Furthermore, a potential application of this anatomical information has already been proposed: the monitoring of the behaviors of APs during acupuncture treatment by measuring electroactive molecules, such as serotonin, secreted by granules of MCs [24].

**Figure 1:**
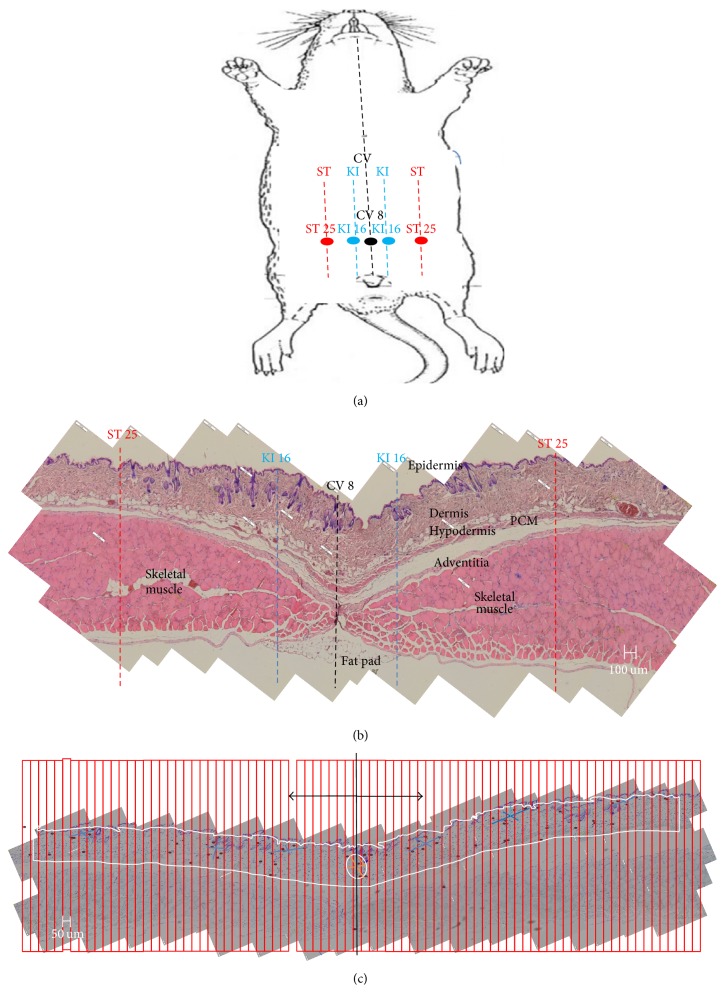
(a) Schematic illustration of acupoints along the meridian in the abdomen of a mouse. Three meridians, conception vessel (CV), kidney (KI) lines, and stomach (ST) lines, are shown. The acupoint CV 8 is located at the umbilicus. (b) Cross-sectional image of a mouse abdomen showing its layers (epidermis, dermis, hypodermis, panniculus carnosus muscle (PCM), adventitia, skeletal muscle, fascia, and fat pad). The traditional locations of acupoints are indicated with dotted lines. (c) A toluidine-blue stained sample was imposed by a mesh of strips (50 *μ*m each) in order to facilitate counting of MCs. The acupoint CV 8 is indicated with an ellipse and the region of background is shown by the enclosing curve.

Because of the significant roles of MCs in both Western medicine and acupuncture, systematic investigations of the characteristics of their distributions in skin so as to extend the findings in previous works would be desirable [4, 5, 18, 19]. For this purpose, the number of MCs in a cross section of skin needs to be determined for the various APs. In this work, we examined a cross section of the abdominal wall. When we tried to observe a sectional view including several APs, we had to be certain not to miss the APs. We found that the APs could easily be missed because the precisions of the locations of the APs were only defined on the order of millimeters in terms of the F-cun method [25]; such a precision was too coarse for histological work involving sections with thicknesses of 5 to 10 *μ*m. Because the umbilicus is one of the most well-defined points in the gross anatomy of abdominal skin and is known as conception vessel 8 (Shenque, CV 8), we chose it as the reference point for our histological work. As shown in Figure 1, two other APs, the kidney 16 (Huangshu, KI 16) and the stomach 25 (Tianshu, ST 25) APs, are located at half F-cun and two F-cun, respectively, from CV 8 along the transversal line. Consequently, for our study, we chose a transverse section of the abdominal wall across the CV 8 acupoint.

## 2. Materials and Methods

### 2.1. Animal Preparation and Sample Taking

For this investigation, 10 male 8-week-old ICR mice were obtained from Young Bio (Seoul, Korea). They were kept in an air-conditioned room at constant temperature and relative humidity (23°C and 60%, resp.) with a 12-hour/12-hour natural light/dark cycle and ad libitum access to food and water. The animals were handled according to current international laws and policies (*Guide for the Care and Use of Laboratory Animals*, National Academy Press, 1996), and the care of the animals and the procedures used in this research were approved by the Institutional Ethics Committee of the Advanced Institute of Convergence Technology, Seoul National University (approval number: WJIACUC20160722-3-01).

The mice were anesthetized by using an intramuscular injection of a regimen consisting of 1.5 g/kg urethane and 20 mg/mL xylazine. The volume of anesthesia administered to the mice was 0.04 mL. All surgery was performed under deep anesthesia, and every effort was made to minimize suffering. The mice were sacrificed by overanesthetizing without any perfusion.

The anterior abdominal wall was removed from the mouse through an incision from immediately below the xiphoid cartilage to the bottom line of the urinary bladder so as to include either side of the right and the left superficial epigastric vessels bilaterally. We removed the specimen and washed it for an hour in tap water, after which it was immediately placed in a 10% neutral buffered formalin (NBF) solution and stored for one day at room temperature. We also used a second fixative called Orth's solution, which contains potassium dichromate. In this procedure, after Orth's fixation for a minimum of 24 hours, the specimens had to be stored in 70% ethyl alcohol (EtOH) until until the surface of the specimen showed any signs of the Orth-brown pigment.

After the fixation steps, we used a razor blade to remove the skin sample from a region ranging from about 500 *μ*m above to 500 *μ*m below the umbilicus and sectioned it transversally to permit wider access to the regions of the CV 8 and the right and the left KI 16 APs bilaterally. These acupuncture points were determined based on the traditional finger-cun measurement procedure corresponding to that for humans. All observations and operations were performed under a stereomicroscope (SZX12, Olympus, Japan).

### 2.2. Staining

In the first fixing method, the isolated abdominal wall specimens were fixed immediately with NBF at 23°C for 24 hr ± 5 hr. The specimens gathered were processed in an automated tissue processor, after which they were embedded in paraffin wax. The resulting formalin-fixed paraffin-embedded blocks were cut into 5 *μ*m thick sections by using a microtome (Reichert Jung 820, Leica, Germany). The sectioning continued until the search area, the middle parts of the umbilicus region (CV 8), had been reached. In the second fixing method, the isolated abdominal wall samples were immediately fixed with Orth's fixation solution at 23°C for 24 hours while avoiding exposure to light. After fixation, the samples were washed in running water overnight and were then stored in 70% EtOH until use.

Two consecutive paraffin sections of 5 microns each from the search point were cut from the paraffin block. One was stained using the conventional hematoxylin and eosin (H&E) staining method and the other section was stained using the toluidine-blue staining method. We performed the H&E staining following a conventional procedure for the purpose of identifying the general histological features of the specimen. For the toluidine-blue staining, the toluidine-blue stock solution was made by melting 0.1 gm of toluidine-blue powder (toluidine-blue O, 198161-5G, Sigma-Aldrich, St. Louis, MO, USA) and 10 mL of 70% alcohol. The working solution at pH 2.3 was made by mixing the stock solution with sodium chloride (1%, pH 2.3). The specimens were stained with toluidine-blue for 60 ± 20 sec. They were dehydrated by dipping them quickly 10 to 15 times first in 95% ethanol and then in 100% ethanol. They were then dipped in xylene for 3 minutes, after which they were mounted on a glass slide.

### 2.3. Counting of Mast Cells

#### 2.3.1. Finding the High-Density Area

The stained specimens were observed under a phase contrast microscope (BX51, Olympus, Japan) to count the MCs, which were easily recognizable because of their stained red-purple (metachromatic staining) color and the background's blue color. The granules from the MCs, which were often scattered around the MCs, were a prominent signature of the MCs. As described by earlier researchers, the distribution of dermal MCs was uneven.

A mechanical calculation of the density of mast cells in dermal connective tissue could have easily led to an erroneous value because of various deformations of the skin, such as shrinkage and distortion, which occurred during the preparation of the sample [18, 19]. Therefore, the counting had to be defined specifically to fit the purpose of the work. In the present work, we divided the abdominal wall into three layers, as shown in Figure 1(c). The 1st layer was the skin from the epidermis to the adventitia of the skeletal muscle. That layer included the dermis and the hypodermis, and the MCs were found to populate this skin layer mostly. The 2nd layer was from the adventitia to the fascia underneath the abdominal muscle. The 3rd layer was the fat tissue below the muscle layer. The fat tissue formed a pad directly below the umbilicus; MCs were also observed in that pad.

**Figure 2:**
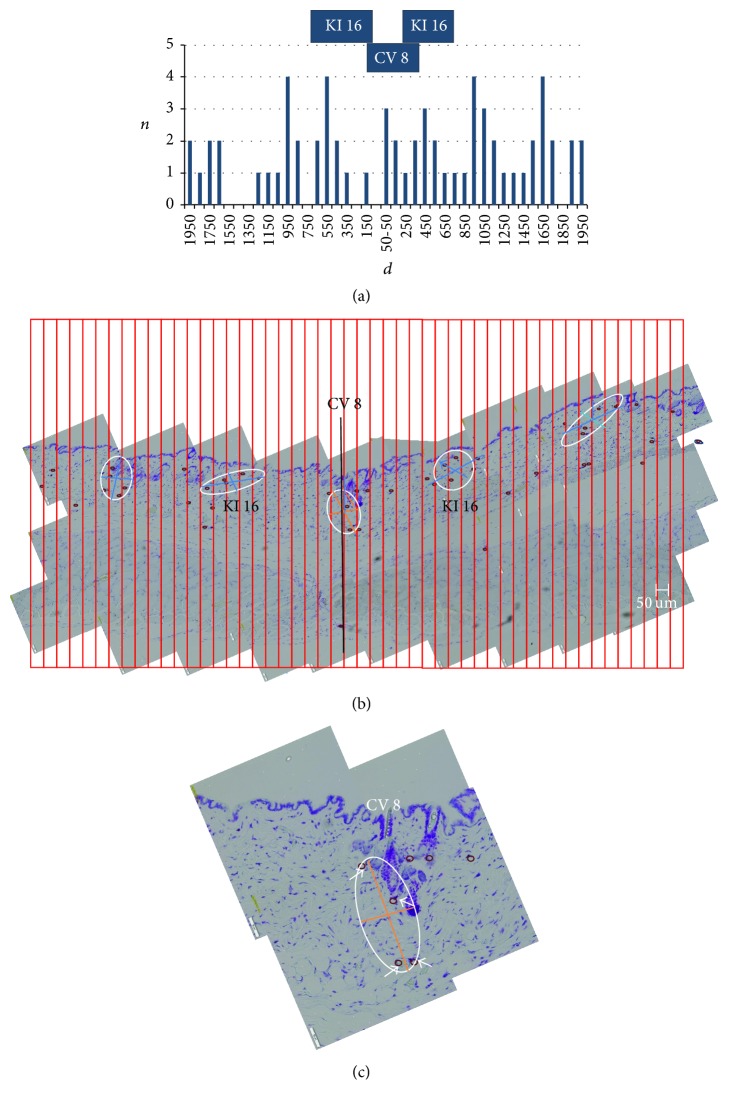
(a) Graphical representation of MC density from Figure 1(c). *X*-axis (*d*) is the distance from CV8 in unit of *μ*m. *Y* axis (*n*) is the number of MCs in 100 *μ*m. (b) The same image of Figure 1(c) showing the selected five regions of high density of MCs with ellipses. The putative acupoints CV 8 and right and left KI 16 are indicated. Two more high-density points are also shown. They might correspond to ST 25 s. (c) A magnified view of the region of CV 8 in Figure 1(c). Four MCs are indicated with arrows. The center and the long and short axes of the covering ellipse are location and size of CV 8, respectively. The center lied in deep dermis below a hair follicle.

MC counts per length, instead of area, were considered by Larsson and Sylven as a practical way of density comparison [18], but we modified that method to apply a mesh of 50 *μ*m strips to the sample, as shown in Figure 1(c). These narrow strips helped experimenters to count the MCs correctly without missing any or overcounting them. The MC counts of two consecutive 50 *μ*m strips were recorded and presented graphically in Figure 2(a) in the form of histograms.

#### 2.3.2. Calculating the MC Number Density at the APs in the Skin and for the Background

Having approximately found the high-density regions by using the histograms, we calculated more precisely the number density of MCs by measuring the area of each region by drawing, as shown in Figure 2(b), an ellipse that covered the MCs. Instead of the density at nonacupoints, the overall background average density throughout the entire skin of the dermis and the hypodermis, excluding the high-density regions, was calculated. For this, we used ImageJ and the TSView programs and counted the numbers of pixels. The size of a pixel was 0.64 × 0.64 *μ*m^2^. The numbers of MCs, the areas of the high-density regions, and the number densities are presented in Table 1. The average density and other quantitative data were expressed as means ± standard deviations for ten animals. The locations of the putative APs, the CV 8 and the left and the right KI 16 APs, as determined from the densities of MCs, were given as the centers of the ellipses that covered the high-density areas. The locations, depths, and sizes of these putative APs are presented in Table 2. The average was taken over ten mice, and the standard deviations of the error are given.

## 3. Results

MCs were mostly distributed in the dermis and the hypodermis but were never found in the epidermis. Only very few MCs were observed near blood vessels below the hypodermis, that is, in the layers of the adventitia, skeletal muscle, and fascia. Surprisingly, MCs again occurred in the fat pad underneath the fascia, a finding that was not reported in previous studies [18, 19]. From the graphs in Figure 2(a) showing the MC distributions, one can clearly see that the distributions are not even. From this gross feature of the distributions we selected regions that should be examined more closely. Figure 2(b) shows the five selected regions of abundant MCs.

We examined the locations of the high-density regions in connection with the three APs, the CV 8 and the right and the left KI 16 APs. In Table 1, we summarize data for the MC densities at the three putative APs and for the background. The average MC number densities were 3.3 ± 1.7/(100 *μ*m)^2^, 2.7 ± 0.9/(100 *μ*m)^2^, and 2.9 ± 0.9/(100 *μ*m)^2^ at the CV 8 and the right and the left KI 16 acupoints, respectively, where we have used the practical and convenient unit of area for experimenters, that is, 100 *μ*m × 100 *μ*m. The MC number density of the background was 0.3 ± 0.1/(100 *μ*m)^2^. The area and the MC density at the fat pad were 11.8 ± 9.9 × 10^4^ *μ*m^2^ and 0.6 ± 0.5/(100 *μ*m)^2^, the latter being about two times higher than the background density of the dermis and the hypodermis.

In Table 2, the locations, that is, the distances from the umbilicus, the depths from the epidermis, and the sizes of the long and the short axes of the three APs are recorded. The distances from the CV 8 to the right and the left KI 16 APs were 541.8 ± 184.8 *μ*m and 565.4 ± 178.4 *μ*m, respectively. The depths were 193.6 ± 97.0 *μ*m, 163.4 ± 64.2 *μ*m, and 135.7 ± 69.7 *μ*m for the CV 8 and the right and the left KI 16 APs, respectively. The centers of the APs were mostly in the deep dermis and sometimes in the upper hypodermis. The average long axis × the short axis for the CV 8 AP was 157.9 ± 62.1 *μ*m × 90.0 ± 17.5 *μ*m. Similarly, for the right and the left KI 16 APs, they were 178.9 ± 54.2 *μ*m × 108.3 ± 32.2 *μ*m and 173.1 ± 49.4 × 107.0 ± 29.8 *μ*m, respectively.

The region corresponding to the CV 8 AP is shown in Figure 2(c). The area and the MC count in it were 2.6 × 10^4^ *μ*m^2^ and 4, respectively, giving a number density of 1.5/(100 *μ*m)^2^. This region was enclosed by an ellipse with long and short axes of 283 *μ*m and 117 *μ*m, respectively. This can be considered as a gross anatomical description of the putative CV 8 AP.

**Figure 3:**
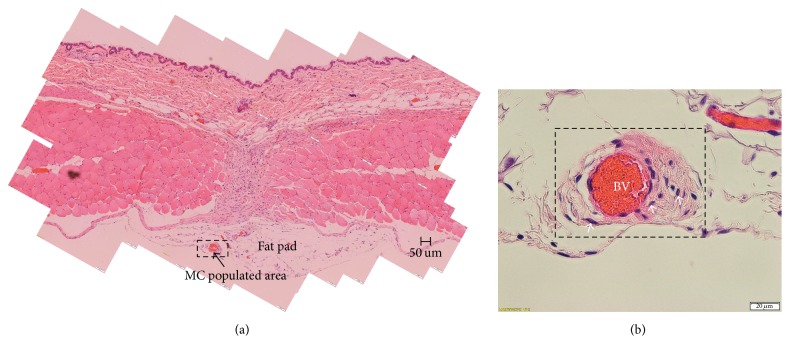
(a) An H&E image showing the fat pad under the abdominal wall muscle. The MC populated region is indicated with a box. (b) A magnified view of the boxed region of (a). Three MCs near a blood vessel (BV) are indicated with arrows.

Figure 3 shows a fat pad with a diameter. MCs were observed near the blood vessels in this fat pad. However, MCs of other mice were not in the similar location, sometimes being near the fascia and other times being near the parietal peritoneum. The distribution was not left-right symmetric either.

## 4. Discussion

Previous studies [4–8] on the relation between MCs and APs considered traditionally defined APS and they found higher densities of MCs at APs than neighboring non-APs. However, they did not investigate whether only APs have many MCs or there are non-APs which still have high density of MCs. Logically speaking, they found that the high MC density is a necessary condition of traditional APs but did not know whether it is also a sufficient condition. In this work, we found that it is not a sufficient condition because there are many more points of high MC density compared to traditionally given APs. This new finding was possible because we investigated the MC distribution throughout the whole skin section, whereas previous studies examined only some preselected points. These high MC density non-APs raise new subjects for acupuncture study: whether they are hitherto unknown novel APs or merely accidental coincidences. If the former is right, MCs could be used to find new APs. If the latter holds, it remains to find the common factors that gather many MCs in APs and non-APs. In addition, and more importantly, our method of studying the MC density throughout the whole skin has the advantage of providing quantitative information about the locations, depths, and sizes of the APs up to the submillimeter scale which was not given in previous studies [4–8]. This cytological method is a newly developed tool which is useful for characterizing the APS together with conventionally used anatomical and histological methods [20, 25, 26].

Our results are in agreement with the first quantitative analysis of the distribution of MCs in the skin of a mouse by Larsson and Sylven [18], despite the uses of different strains of mice (ICR versus Swiss albino) and different locations. We studied the ventral skin near the umbilicus, while they studied the interscapular regions of the dorsal skin. Both teams found a high density of MCs in the deep dermis and hypodermis, no MCs in the epidermis, and only very few in the adventitia, skeletal muscles, and fascia. These results are also consistent with data for the human arm [19].

In our case, the average background number density of MCs in the deep dermis and hypodermis for abdominal skin was 0.3 ± 0.1/(100 *μ*m)^2^. Larsson and Sylven reported that it was 2.4/(100 *μ*m)^2^ in the dermis and 0.74/(100 *μ*m)^2^ in the hypodermis [18]. Similarly, the MC density at nonacupoints near some APs of rats was reported to be 1.3~1.8/(100 *μ*m)^2^ [6]. In the case of human skin, Eady et al. reported 0.47/(100 *μ*m)^2^ [19], and Craig and Schwarz quoted 0.31/(100 *μ*m)^2^ in the dermis [27]. Large variations in the MC densities are known to exist, depending on the species, the individual, and the location in the skin. When we consider the different histological processes, different positions, and individual variations in the various studies, we can conclude that our number densities are in reasonable agreement with those published elsewhere. In addition, a mechanical calculation of the density of mast cells in dermal connective tissue may easily lead to different values because of the various deformations of the skin which can occur during the preparation of the sample [18, 19]. Therefore, the density should not be taken as a precise standard average value, which may not even be definable.

Nevertheless, regions having clumped high densities of MCs, which are the putative APs, were consistently observed and were in accord with previous results for rats [4, 5]. In this study, the average MC number densities were 3.3 ± 1.7/(100 *μ*m)^2^, 2.7 ± 0.9/(100 *μ*m)^2^, and 2.9 ± 0.9/(100 *μ*m)^2^ at the CV 8 and the right and the left KI 16 APs, respectively, which are in agreement with the data for rats: 2.3 ± 0.7/(100 *μ*m)^2^ at ST 36, 2.4 ± 0.6/(100 *μ*m)^2^ at ST 31, and 2.3 ± 0.6/(100 *μ*m)^2^ at ST 25 [6].

In the current work, we were able to infer the locations, depths, and sizes of the three APs, CV 8 and the left and the right KI 16 APs, by making use of the number density of MCs. The MCs were all in the dermis and the hypodermis and had oval shapes with sizes of about 200 *μ*m, as shown in Table 2. The distance of the CV 8 AP from the umbilicus was 70.9 ± 44.9 *μ*m. The distances of the right and the left KI 16 APs from CV 8 were 541.8 ± 184.8 *μ*m and 565.4 ± 178.4 *μ*m, respectively. These correspond to 0.4 ± 0.1 F-cun. We used the F-cun measurement of finger width [25]. In our case, 1 F-cun was approximately 1.3 mm. The F-cun measure of the KI 16 AP is 0.5 in traditional knowledge.

However, the regions highly populated with MCs did not always coincide with conventionally designated APs. For example, the next points from the KI 16 APs were only about 1,300 *μ*m away from CV 8, as shown in Table 2. If they were ST 25 as in traditional Chinese medicine, the distance should be about 2,600 *μ*m. The extra high MC density point at 1,300 *μ*m might be a novel extra AP which does not exist in a human body. If this is true in general the MC density can be a novel cytological method to find extra APs. However, it might not be an AP at all. In this case, a subject to investigate in the future is the reason why some non-APs have high-density MCs. Currently the reason is not known either in traditional Chinese medicine or in Western medicine.

One obvious limitation of the current work is the failure to determine the boundaries enclosing the APs by using the MC distribution. The reason was the limitation of the staining dye toluidine-blue that shows MCs well but cannot show many other components of the skin tissue. At the present time, the dye to stain the boundary cells or tissues of the APs is not known. For example, Hemacolor technique stained many other components of the primo node but it did not specifically show the boundary tissue [22]. So, it remains an important task to find the right dye to stain the boundary tissue of the APs, which we hope to solve in the future.

Interestingly, we found that rats and mice are different with respect to the fat tissue underneath the fascia. The fat pad of a mouse was a round or oval disk shape of about 1,000 *μ*m in diameter (Figure 3(a)). A rat has a long fat band, as compared to the fat pad of a mouse. Both the long fat band in a rat and the fat pad in a mouse are populated with MCs. The fat band of a rat has a PVS in it, and high densities of MCs in the PVS have recently been found in several works on the primo nodes in the abdominal cavity [9, 21–23].

The methods used in this study can be used to investigate the distributions of MCs for other skin areas to determine the gross anatomical features of APs. Similar studies on pathological conditions in mice will be useful for investigating the roles of MCs in the treatment of diseases. One medically significant application of nanotechnology to detect and monitor ST 36 by measuring the serotonin secreted by the MCs residing in the APs was proposed by Li et al. [24]. For this purpose, knowledge of both the locations and the depths of the APs, for which the current work provides useful information, is essential.

## Figures and Tables

**Table 1:** Number density of mast cells at the putative acupoints CV 8 and KI 16 and at the fat pad and the background density.

*M*	*A*/*W*	CV 8			Right KI 16			Left KI 16			Right NHP			Left NHP			B			Fat pad		
		*N*	*A*	*D*	*N*	*A*	*D*	*N*	*A*	*D*	*N*	*A*	*D*	*N*	*A*	*D*	*N*	*A*	*D*	*N*	*A*	*D*
1	8/26	4	2.6	1.5	7	1.8	3.9	5	2.3	2.2	6	1.9	3.2	6	2.7	2.2	33	83.1	0.1	1	2.5	0.4
2	8/25	4	1.4	2.9	5	1.8	2.8	3	1.4	2.1	6	2.3	2.6	3	2.7	1.4	28	278.6	0.1	2	33.3	0.1
3	8/25	2	0.7	2.9	3	1.4	2.1	4	1.3	3.1	4	1.6	2.5	6	1.4	4.3	30	132.1	0.2	7	13.8	0.5
4	8/26	10	1.3	7.7	4	2.1	1.9	3	0.9	3.3	4	2	2	4	1.4	2.9	40	81.7	0.5	5	8.6	0.6
5	8/26	2	0.9	2.2	4	1.2	3.3	2	0.6	3.3	6	1.3	4.6	3	1.1	2.7	43	153.8	0.3	4	9.8	0.4
6	8/27	3	1.8	1.7	3	3.5	0.9	4	3.1	1.3	4	3	1.3	4	4	1	33	105.1	0.3	5	26.1	0.2
7	8/24	5	1.7	2.9	3	1	3	7	2.7	2.6	3	1.8	1.7	3	5.6	0.5	26	119.6	0.2	1	0.8	1.3
8	8/27	2	0.5	4	2	0.5	4	2	0.8	2.5	4	1.0	4	5	3.9	1.3	5	99.2	0.1	3	10.3	0.3
9	8/26	3	0.8	3.8	2	0.7	2.9	5	1	5	4	1.6	2.5	7	1.6	4.4	37	162.8	0.2	4	9	0.4
10	8/24	2	0.6	3.3	4	1.7	2.4	4	1.3	3.1	5	2.2	2.3	3	1.7	1.8	40	76.2	0.5	6	3.5	1.7
Average	8/25.6	3.7	1.2	3.3	3.7	1.6	2.7	3.9	1.5	2.9	4.6	1.9	2.7	4.4	2.6	2.3	31.5	129.2	0.3	3.8	11.8	0.6
SD	0/1.0	2.3	0.7	1.7	1.4	0.8	0.9	1.4	0.8	0.9	1.0	0.5	1.0	1.4	1.5	1.3	10.3	57.3	0.1	1.9	9.9	0.5

*M*  = mouse number; *A*/*W*  = age (week)/weight (g); NHP = next high MC density point; B = background; *N*  = number of MCs; *A*  = area (10^4^ um^2^); *D*  = *N*/*A*  (number/10^4^ *μ*m).

**Table 2:** Locations, depths, and sizes of the putative acupoints CV 8 and KI 16.

*M*	*A*/*W*	CV 8			Right KI 16			Left KI 16			Right NHP			Left NHP		
		*X*_*c*_	*Z*	*L*/*S*	*X*	*Z*	*L*/*S*	*X*	*Z*	*L*/*S*	*X*	*Z*	*L*/*S*	*X*	*Z*	*L*/*S*
1	8/26	+114	250	283/117	416	177	208/113	443	88	245/119	964	133	233/105	883	164	293/118
2	8/25	−50	182	152/117	783	113	233/96	712	79	154/115	1467	383	258/115	1430	85	163/163
3	8/25	+17	25	128/67	450	129	203/89	550	194	167/100	1190	325	207/99	1767	258	144/128
4	8/26	+175	50	195/85	588	183	165/165	683	283	143/80	1523	250	218/119	1126	300	236/77
5	8/26	−67	133	133/83	356	64	180/82	318	137	90/86	922	112	149/111	1393	58	123/114
6	8/27	+50	317	228/98	483	233	275/163	580	67	220/179	1445	70	285/132	1617	233	250/202
7	8/24	+25	153	238/92	673	222	175/76	771	219	254/133	1634	171	174/132	1445	219	320/222
8	8/27	−44	290	116/60	888	220	83/83	738	111	137/73	1206	102	130/101	1208	109	265/187
9	8/26	−67	290	105/94	525	233	100/83	209	62	142/90	1188	112	208/99	939	56	183/110
10	8/24	+100	246	88/87	256	60	167/133	650	117	179/95	783	167	230/123	1251	200	214/102
Average	8/25.6	70.9	193.6	157.9/90	541.8	163.4	178.9/108.3	565.4	135.7	173.1/107	1232.2	182.5	209.2/113.6	1305.9	168.2	219.1/142.3
SD	0/1.0	44.9	97.0	62.1/17.5	184.8	64.2	54.2/32.2	178.4	69.7	49.4/29.8	268.5	98.5	45.1/12.1	266.5	82.7	61.9/45.7

*M*  = mouse number; *A*/*W*  = age (week)/weight (g).

*X*_*c*_  = distance from the navel (*μ*m) in CV 8; *X*  = distance from CV 8 to KI 16 acupoints and NHPs, respectively.

*Z*  = depth from the epidermis (*μ*m).

*L*/*S*  = long axis/short axis of the covering ellipse (*μ*m).
